# Comparison of the effects of intrauterine and rectal misoprostol combined with oxytocin versus oxytocin alone on postpartum hemorrhage: A randomized controlled trial

**DOI:** 10.1097/MD.0000000000047091

**Published:** 2026-01-02

**Authors:** Gökhan Güler, Onur Karaaslan

**Affiliations:** a Department of Obstetrics and Gynecology, Van Training and Research Hospital, Van, Turkey; b Department of Obstetrics and Gynecology, Van Yuzuncu Yil University, Van, Turkey.

**Keywords:** intrauterine misoprostol, oxytocin, postpartum hemorrhage

## Abstract

**Background:**

This study aimed to compare the effects of intrauterine and rectal misoprostol administration in addition to oxytocin, versus oxytocin alone, in preventing postpartum hemorrhage (PPH).

**Methods:**

A total of 150 patients scheduled for cesarean delivery and considered at risk for PPH were included in the study. The patients were divided equally into 3 groups: those who received only oxytocin (Group 1), those who received rectal misoprostol in addition to oxytocin (Group 2), and those who received intrauterine (cornual) misoprostol along with oxytocin (Group 3). The groups were compared in terms of demographic, preoperative, intraoperative, and postoperative characteristics, laboratory findings, amount of intrauterine fluid, and infection parameters.

**Results:**

No statistically significant differences were observed between the groups in terms of age, obstetric history, preoperative hemoglobin (Hb) and hematocrit (Htc) values, systolic-diastolic blood pressures, and intraoperative pad counts (*P* > .05). Hb and Htc levels recorded at postoperative 4th and 24th hours, and 10th day were statistically significantly higher in Groups 2 and 3 compared with Group 1 (*P* < .001). While the total aspirator fluid (TAF) amount was similar between Groups 1 and 2, the TAF amount was statistically lower in Group 3 (*P* < .01). The number of intraoperative sponges and the mean volume of intracavitary fluid loculation were statistically significantly lower in Groups 2 and 3 compared with Group 1 (*P* < .01). Intraoperative sponge count and mean intracavitary fluid loculation volume in Group 3 were statistically significantly lower than in Group 2 (*P* < .01). No difference was observed between mean white blood cell values, and no statistically significant difference was observed between mean C-reactive protein (CRP) values preoperatively and 24 hours postoperatively. On the 10th postoperative day, the mean CRP value in Group 3 was statistically lower than in Group 1 (*P* = .06).

**Conclusion:**

In cesarean deliveries with high risk of PPH, the addition of intrauterine (cornual) misoprostol to oxytocin administration may provide better outcomes in terms of reducing postpartum bleeding and infection risk.

## 1. Introduction

Postpartum hemorrhage (PPH) is one of the most significant causes of maternal morbidity and mortality after childbirth and is a leading cause of maternal death, particularly in the countries with low and middle-income.^[1]^ It accounts for approximately 25% of direct maternal deaths worldwide.^[2]^ PPH not only leads to maternal death but can also lead to serious complications such as hypovolemic shock, disseminated intravascular coagulation, hysterectomy, infertility, infection, and prolonged hospitalization.^[3]^

The most common cause of PPH is uterine atony, which occurs due to inadequate uterine contraction after childbirth.^[3]^ Therefore, the use of uterotonic medications within the context of active labor management has become a fundamental strategy for preventing PPH. Organizations such as the American College of Obstetricians And Gynecologists, the International Federation of Gynecology and Obstetrics, and the World Health Organization recommend intravenous (IV) or intramuscular oxytocin as the first-line prophylactic agent after childbirth.^[4–7]^ Despite its short half-life, oxytocin has a potent uterine contraction effect and its effects appear rapidly. However, in some cases, its effect may be insufficient, and additional agents are required, particularly in high-risk pregnancies.^[8]^

At this point, misoprostol, a prostaglandin E1 analog, stands out with its uterotonic efficacy, low cost, ability to be stored at room temperature, and various routes of administration (oral, sublingual, rectal, vaginal, and intrauterine).^[9]^ Misoprostol is also listed by the World Health Organization as a drug for the prevention and treatment of PPH, and it has been reported that maternal deaths due to bleeding in underdeveloped countries, particularly after misoprostol use, have decreased.^[10]^ In addition to its systemic effects, misoprostol has been shown to directly increase uterine contractility through its local application. However, studies comparing different routes of administration of misoprostol are limited in number and have limitations, creating uncertainties in clinical decision-making.^[11–13]^

Rectal misoprostol, despite its slower absorption and lower bioavailability, is often preferred due to its noninvasive nature. In both contractile and electrophysiological experiments, no specific pacemaker site was found where uterine contractions initiated. However, some rat experiments have identified uterine contractions originating in the cornual region near the ovary.^[14]^ Therefore, it is thought that misoprostol administered intrauterine directly to the cornual region may be more rapid and effective. Furthermore, such local administration is thought to have the potential to reduce systemic side effects.

Although there are many studies in the literature on the use of misoprostol by oral, sublingual, vaginal and rectal routes, the number of randomized controlled trials directly evaluating the effectiveness of intrauterine-cornual administration is quite low.^[15]^

In this study, we aimed to compare the effectiveness of rectal misoprostol and intrauterine misoprostol combined with oxytocin in preventing PPH compared to oxytocin alone in pregnant women with a planned cesarean section and at high risk for PPH.

## 2. Material & method

This study was planned as a prospective, randomized controlled clinical trial. The aim of the study was to compare the effects of oxytocin alone and oxytocin combined with rectal or intrauterine (cornual) misoprostol on postpartum bleeding in pregnant women scheduled for cesarean delivery and at high risk for PPH. The study was conducted at the Van Yuzuncu Yil University Obstetrics and Gynecology Clinic between July 1, 2023, and January 1, 2024. Approval for the study was received from the Interventional Ethics Committee of Van Yuzuncu Yil University, Faculty of Medicine (Date: May 31, 2023, No: 14). All participants were provided detailed information about the study’s purpose, methods, potential side effects, and confidentiality principles. Written informed consent was obtained, and the principles of the declaration of Helsinki were adhered to throughout the study.

### 2.1. Participant selection

#### 2.1.1. Inclusion criteria

Pregnant women in the 18 to 45 age range, Pregnant women ≥ 37 weeks gestation, Patients scheduled for elective cesarean section, Those with at least one risk factor for PPH (e.g., macrosomia, polyhydramnios, multiple pregnancy, advanced maternal age, uterine fibroids, history of PPH).

#### 2.1.2. Exclusion criteria

Obstetric conditions requiring emergency cesarean section (e.g., fetal distress, placental abruption), Known allergy to uterotonic agents, Coagulopathy or bleeding diathesis, Use of anticoagulant therapy, Active infection or febrile illness, Severe cardiovascular, hepatic, or renal disease, Patients who refuse informed consent, Hemoglobin (Hb) < 10 mg/dL, Hypertensive disorders of pregnancy (e.g., preeclampsia, eclampsia, HELLP, etc).

### 2.2. Sample size and randomization

Sample size calculations were based on previous similar studies, with α = 0.05 and power = 0.90. A minimum of 40 individuals/groups was deemed sufficient, and 50 patients were included in each group, taking into account potential attrition. A total of 150 patients were included in the study. Randomization was performed using a computer-assisted block randomization method.

#### 2.2.1. Group 1 (control group, n = 50)

Within the first hour after umbilical cord clamping, 20 IU/500 mL of isotonic fluid was administered as an infusion.

#### 2.2.2. Group 2 (rectal misoprostol group, n = 50)

In addition to the 20 IU/500 mL of isotonic fluid infusion within the first hour after umbilical cord clamping, 600 mcg of misoprostol was administered rectally.

#### 2.2.3. Group 3 (intrauterine misoprostol group, n = 50)

Within the first hour after umbilical cord clamping, 20 IU/500 mL of isotonic fluid was administered as an infusion. 600 mcg of misoprostol was placed into the intrauterine cornual region.

### 2.3. Data collection and monitoring process

#### 2.3.1. Preoperative

Demographic data: age, number of pregnancies, number of births, and gestational age were recorded.

Vital signs: systolic/diastolic blood pressure, pulse, and laboratory data: Hb, hematocrit (Htc), white blood cell (WBC), C-reactive protein (CRP), and platelet values were recorded.

#### 2.3.2. Intraoperative

Number of sponges and pads used, amount of total aspirator fluid (TAF).

#### 2.3.3. Postoperative

Postoperative 0 to 4th hour: Number of patient diapers (chux pads); Postoperative 4th hour: Hb and Htc levels, WBC count; Postoperative 24th hour: Hb, Htc and CRP levels, WBC count; Postoperative 10th day: Hb, Htc and CRP levels, WBC count, mean intracavitary fluid volume (length × depth × width × 0.52).^[16]^

### 2.4. Statistical analysis

The analysis of the study was conducted using the SPSS 26.0 package program. The conformity of numerical variables to normal distribution was assessed using the Kolmogorov–Smirnov test. Mean ± standard deviation values were presented for summarizing continuous numerical variables, while numbers and percentages were presented for summarizing categorical variables. In comparing continuous numerical variables among 3 independent groups, the Kruskal–Wallis test was used when a normal distribution was not achieved, and one-way analysis of variance (ANOVA) was used when a normal distribution was achieved. Bonferroni correction was applied to determine the group from which the detected significance originated, and pairwise comparison was used for non-normally distributed data and the post hoc Tukey test was used for normally distributed data. *P* value of <.05 was considered statistically significant.

## 3. Results

A total of 150 pregnant women were included in the study. All participants were equally distributed into 3 groups (n = 50) after randomization: Group 1 (n = 50): Those receiving IV oxytocin alone; Group 2 (n = 50): Those receiving oxytocin + rectal misoprostol (600 mcg); Group 3 (n = 50): Those receiving oxytocin + intrauterine (cornual) misoprostol (600 mcg).

No statistically significant differences were observed between the groups in terms of age, obstetric characteristics, or vital signs (*P* > .05), (Table 1).

**Table 1:** Comparison of age, obstetric parameters and vital signs of the groups.

Variables	Groups			
	Group 1 (n = 50)	Group 2 (n = 50)	Group 3 (n = 50)	*P*
	Mean ± SD	Mean ± SD	Mean ± SD	
Age (yr)	30.26 ± 5.24	31.14 ± 5.84	28.7 ± 4.36	.062
Gravidity	2.78 ± 1.31	2.94 ± 1.11	2.58 ± 1.25	.403
Parity	2.74 ± 1.29	2.92 ± 1.12	2.58 ± 1.25	.428
VB	0.42 ± 1.05	0.38 ± 0.9	0.14 ± 0.4	.522
C/S	1.34 ± 1.32	1.56 ± 1.25	1.46 ± 1.33	.675
SBP (mm Hg)	118.6 ± 7.07	118.5 ± 4.55	119.1 ± 5.02	.814
DBP (mm Hg)	74.4 ± 6.75	75.9 ± 4.67	75 ± 4.95	.459

C/S = ceserean section, DBP = diastolic blood pressure, *P* = <.05, SBP = sistolic blood pressure, SD = standard deviation, VB = vaginal birth.

While no statistically significant difference was found between the groups in terms of preoperative Hb values (*P* = .322), when compared with Group 1 in terms of Hb levels measured at postoperative 4th hour, 24th hour and 10th day, the mean Hb values of Group 2 and Group 3 were observed to be statistically higher (*P* < .01). No statistically significant difference was observed in the Hb values between Group 2 and Group 3 (*P* > .05). While no statistically significant difference was observed between the preoperative and postoperative 24th hour CRP values, the mean CRP value of Group 3 on the postoperative 10th day was observed to be statistically lower than Group 1 (*P* = .06). When looking at the WBC values, no statistical difference was observed between the preoperative, postoperative 4th hour, 24th hour and 10th day values (*P* > .05), (Table 2).

**Table 2:** Comparison of hemoglobin values recorded at different times between groups.

	Groups			
Laboratory parameters	Group 1 (n = 50)	Group 2 (n = 50)	Group 3 (n = 50)	*P*
	Mean ± SD	Mean ± SD	Mean ± SD	
Hb (g/dL) preoperative	12.36 ± 1.11	12.68 ± 0.85	12.48 ± 1.18	.322
Hb (g/dL) PO 4th hour	10.87 ± 1.14*	11.71 ± 0.78†	11.55 ± 1.12†	<.01
Hb (g/dL) PO 24th hour	10.12 ± 1.12*	11.3 ± 0.87†	11.16 ± 1.11†	<.01
Hb (g/dL) PO 10th day	10.85 ± 1.24*	11.68 ± 0.81†	11.73 ± 1.07†	<.01
CRP preoperative	6.5 ± 1.23*	6.14 ± 1.05*	6.64 ± 1.24	.345
CRP PO 24th hour	75.88 ± 26.71	83.38 ± 25.81	76.46 ± 20.99	.241
CRP PO 10th day	27.74 ± 18.91*	25.46 ± 9.85	17.62 ± 8.01†	.06
WBC preoperative	10,36 ± 1,91	9,71 ± 2,14	10,48 ± 1,84	.109
WBC PO 4th hour	14.67 ± 3.03	14.25 ± 2.92	15.04 ± 2	.101
WBC PO 24th hour	12.35 ± 2.52	13.0 ± 2.93	12.89 ± 1.84	.438
WBC PO 10th day	9.81 ± 2	9.8 ± 1,53	9.44 ± 1.56	.351

CRP = C-reactive protein, Hb = hemoglobin, *P* = <.05, PO = postoperative, SD = standard deviation, WBC = white blood cell.

* Indicates a significant difference between Group 1 and Group 2.

† Indicates a significant difference between Group 1 and Group 3.

Compared with Group 1, the number of intraoperative sponges and the mean volume of intracavitary fluid loculation on postoperative 10th day were statistically significantly less in Group 2 and Group 3. Furthermore, compared with Group 2, the number of intraoperative sponges and the mean volume of intracavitary fluid loculation on postoperative 10th day were statistically significantly less in Group 3 (*P* < .05). There was no statistically significant difference in the number of intraoperative pads between the groups (*P* = .270). In addition, the total postpartum TAF amount and the number of postoperative pads were statistically significantly less in the other 2 groups compared to the oxytocin group (*P* < .01). No statistically significant differences were found in terms of these parameters between Group 2 and Group 3 (*P* > .05), (Table 3).

**Table 3:** Comparison of bleeding characteristics between groups.

	Groups			
Variables	Group 1 (n = 50)	Group 2 (n = 50)	Group 3 (n = 50)	*P*
	Mean ± SD	Mean ± SD	Mean ± SD	
IO number of sponges	14.08 ± 1.35*	13.4 ± 1.28†	12.82 ± 0.9‡	<.01
PO number of pads	8.42 ± 0.95	8.52 ± 0.58	8.3 ± 0.54	.270
PP TAF (mL)	315 ± 39.45*	308 ± 30.9*	288 ± 27.77†	<.01
PO number of pads	3.84 ± 0.62*	3.4 ± 0.53†	3.36 ± 0.48†	<.01
IC liquid amount (mL)	7.12 ± 0.88*	5.99 ± 0.47†	4.35 ± 0.78‡	<.01

IC = intracavitary, IO = intraoperative, PO = postoperative, PP = postpartum, SD = standard deviation, TAF = amount of total aspirator fluid.

* Indicates a significant difference between Group 1 and Group 2.

† Indicates a significant difference between Group 1 and Group 3.

‡ Indicates a significant difference between Group 2 and Group 3.

## 4. Discussion

Despite advances in obstetrics in recent years, PPH remains a leading cause of maternal mortality.^[1,2]^ Most maternal deaths occur within the first 24 hours after birth. Therefore, identifying high-risk patients for PPH and administering appropriate prophylactic treatments to these patients is crucial. Because the majority of PPH (70–80%) occurs due to uterine atony, postpartum prophylactic use of uterotonics can considerably prevent PPH-related deaths.^[3]^ Oxytocin, administered intravenously or intramuscularly followed by an IV infusion, is the primary medical agent used in the first-line treatment of PPH.^[4–7]^ In some cases where oxytocin is insufficient, alternative prophylaxis and treatment options are still being sought. Recent studies have shown that low-dose oxytocin is as effective as high-dose oxytocin in the first-line setting for PPH prevention.^[17]^ It is known that at high doses, the oxytocin receptor undergoes desensitization with agonist exposure.^[17]^ Therefore, secondary uterotonics should be considered earlier. Misoprostol stands out in this regard due to its ease of storage and diverse administration methods. While there are numerous studies in the literature on other forms of misoprostol use, resources regarding its intracavitary use are quite limited.^[15]^

In this study, we compared the efficacy of 3 different protocols administered during cesarean delivery in pregnant women at high risk for PPH: oxytocin alone (Group 1), oxytocin + rectal misoprostol (Group 2), and oxytocin + intrauterine misoprostol (Group 3). Our findings demonstrated that intrauterine (cornual) misoprostol combined with oxytocin was significantly superior in reducing both intraoperative and postoperative blood loss. Rectal misoprostol + oxytocin administration was also found to be advantageous over oxytocin alone, but its efficacy was limited compared to oxytocin + intrauterine misoprostol administration.

Literature indicates that misoprostol alone is less effective than oxytocin in reducing both estimated blood loss and the need for additional uterotonic therapy.^[18]^ Therefore, we planned to use misoprostol alone in combination with oxytocin in a high-risk patient group. In patients with refractory uterine atony – approximately 3 to 25% of patients – other uterotonics are required to increase uterine contractions after oxytocin injection.^[6,19]^ In this case, we planned to use misoprostol in addition to oxytocin due to its easier availability, different routes of administration, and not needing a cold chain to provide it.

Misoprostol can be administered alone when oxytocin is not available during PPH, or it is also recommended in combination with oxytocin. Although there are studies showing that the amount of PPH and the need for blood transfusion are significantly lower in patients using misoprostol alone or in combination with oxytocin compared to those using only oxytocin,^[20–22]^ the presence of studies claiming the opposite suggests that this issue needs to be investigated further.^[11,12]^ In our study, we found that Hb values at 4 hours, 24 hours, and 10 days after cesarean section were significantly higher in Group 2 and Group 3 than in Group 1. The number of intraoperative sponges and postoperative patient diapers (chux pads) used in Group 1 were significantly higher than in Group 2 and Group 3. Furthermore, the postoperative TAF value in Group 3 was found to be statistically significantly lower than in the other 2 groups (*P* < .01).

Pakniat H et al found that the use of low doses of misoprostol-oxytocin significantly reduced blood loss during and after cesarean delivery compared with higher doses of oxytocin or misoprostol alone.^[23]^ These results support the results of our study, regardless of the method of misoprostol administration. Elsedeek MS et al conducted a randomized controlled trial comparing 400-mcg rectal misoprostol (study group) with placebo (control group) immediately after umbilical cord clamping in pregnant women undergoing cesarean delivery.^[24]^ The differences in mean intraoperative blood loss, mean postpartum blood loss, and postoperative Htc values were significantly lower in the study group than in the control group. This study supports our findings, but differently, our study has shown that the use of oxytocin + intrauterine misoprostol, which has been studied in limited numbers on this subject, has better bleeding parameters compared to the use of only oxytocin and oxytocin + rectal misoprostol.

Fawole et al and Hofmeyr et al found no significant reduction in the risk of PPH when misoprostol was used in addition to routine uterotonics for the prevention of PPH.^[11,12]^ However, the misoprostol dose used in this study was limited to 400 mcg. The misoprostol dose in our study was planned as 600 mcg. Considering the results of our study, we think that the 600 mcg dose of misoprostol is effective in preventing PPH regardless of the route of administration. Misoprostol has been used for more than a decade for the prophylaxis and control of PPH after vaginal delivery, but there is no consensus on the optimum dose and best route of administration.^[25]^ When the pharmacokinetic effects of the administration routes were investigated, it was found that the oral route was the easiest to use but had a short duration of action and low bioavailability; the rectal route had slow absorption, low peak level, long duration of action, and fewer side effects; the buccal or sublingual route appears to have rapid absorption, long duration of action, and the highest total bioavailability.^[25,26]^ It is not recommended for the prophylaxis and treatment of PPH because absorption is thought to be reduced due to vaginal bleeding. Oral administration of 400 to 600 mcg of misoprostol is considered optimal for the prevention of PPH, and sublingual administration of 800 mcg of misoprostol is the route with the most evidence supporting its safety and efficacy for the treatment of PPH.^[27,28]^ A limited number of studies have also demonstrated the effectiveness of intrauterine misoprostol in controlling PPH.^[15,29]^ Another important purpose of our study was to compare the effects of different routes of administration: rectal and intrauterine (cornual) misoprostol combined with oxytocin. While no significant difference was found between rectal and intrauterine administration of misoprostol in terms of the decrease in postoperative Hb and Htc levels and the number of postoperative patient diapers (chux pads), the number of intraoperative sponges, TAF and the amount of intracavitary fluid loculation on the 10th postoperative day were found to be significantly less in the group in which misoprostol was administered intracavitary.

It has been suggested that misoprostol, as an adjunct to oxytocin, could be used for earlier interventions in cases of refractory uterine atony.^[30]^ Our study focused primarily on patients with risk factors for PPH. We believe that the intrauterine route, due to its local effect, will yield better results in both prophylaxis and treatment, and our results support this.

In mammals, coordinated uterine contractions are necessary for fetal delivery as well as for the prevention of PPH. The pregnant uterus is a smooth muscle organ which has its contraction pattern determined by the propagation of electrical impulses, and this electrical activity may be due to one or more pacemakers. Unlike other visceral smooth muscles, the precise location of the impulse within the uterus is unknown, and no precise pacemaker site has yet been identified.^[14,31]^ Lammers and colleagues demonstrated that most contraction initiation sites in rats are closer to the cornuco-ovarian portion of the uterus.^[14]^

In our study, 600 mcg of misoprostol administered intracavitary cornually in addition to oxytocin for PPH prophylaxis was found to be the most successful procedure in reducing bleeding. This may be due to the fact that uterine smooth muscle contractions that reduce PPH after delivery most likely initiate in the cornual region, are concentrated in this region, and may be enhanced by exogenously increased prostaglandins in this region. However, to make definitive interpretations of these results, comparisons of topical misoprostol applied to different regions of the uterus with cornual application and electrophysiological studies are necessary. We hope that our study will inspire future studies on this topic.

Severe PPH has also been associated with longer operative times, longer hospital stays, and an increased risk of infection secondary to hemodynamic changes.^[32]^ In this context, it can be assumed that uterotonic agents may contribute to reducing the risk and severity of PPH and thus reduce the risk of potential infection. In the current study, no statistically significant difference was observed between the groups in terms of WBC values at preoperative, postoperative 4th hour, 24th hour, and 10th day (*P* > .05). No difference was observed between the groups in terms of preoperative and postoperative 24th hour mean CRP values (*P* > .05). Postoperative 10th day CRP levels were statistically lower in Group 3 than in Group 1. There are insufficient studies on the effects of misoprostol on infection and infection parameters in cases where it is used for PPH. In a study comparing the side effects of oral misoprostol with placebo, no statistically significant difference was found between the 2 groups in terms of infection rates.^[33]^ The incidence of infection after the use of misoprostol for medical abortion was reported as 0.92% in one study.^[34]^ In our study, Groups 2 and 3 had less intracavitary fluid on the 10th postoperative day compared to Group 1. Between Groups 2 and 3, the mean intracavitary fluid volume was statistically lower in Group 3. Therefore, the use of intracavitary misoprostol, in particular, suggests that it may reduce the risk of postpartum infection. The statistically lower mean CRP value in Group 3 on the 10th postoperative day supports this view.

Our study has some limitations; because it was conducted at a single center, the generalizability of the results is limited. Because the study population consisted of patients at high risk for PPH, a placebo group could not be included. Comparisons regarding side effects were also not possible.

## 5. Conclusion

In our study, in cesarean deliveries with high risk of PPH, the combined use of misoprostol with oxytocin was shown to have better intraoperative and postoperative bleeding parameters compared to the use of oxytocin alone. Regarding postoperative infection parameters, particularly better results were obtained in the intrauterine misoprostol group. Based on our findings, the use of intrauterine misoprostol is considered to be an effective, safe, and applicable option.

## Author contributions

**Conceptualization:** Gökhan Güler.

**Data curation:** Gökhan Güler.

**Formal analysis:** Gökhan Güler, Onur Karaaslan.

**Investigation:** Onur Karaaslan.

**Methodology:** Gökhan Güler, Onur Karaaslan.

**Validation:** Gökhan Güler, Onur Karaaslan.

**Visualization:** Onur Karaaslan.

**Writing – original draft:** Gökhan Güler, Onur Karaaslan.

**Writing – review & editing:** Gökhan Güler, Onur Karaaslan.
